# Vascular Endothelial Growth Factor Is Regulated by the Canonical and Noncanonical Transforming Growth Factor-*β* Pathway in Synovial Fibroblasts Derived from Osteoarthritis Patients

**DOI:** 10.1155/2019/6959056

**Published:** 2019-09-29

**Authors:** Shotaro Takano, Kentaro Uchida, Shintaro Shoji, Makoto Itakura, Dai Iwase, Jun Aikawa, Manabu Mukai, Hiroyuki Sekiguchi, Gen Inoue, Masashi Takaso

**Affiliations:** ^1^Department of Orthopedic Surgery, Kitasato University School of Medicine, 1-15-1 Minami-ku, Kitasato, Sagamihara City, Kanagawa 252-0374, Japan; ^2^Department of Biochemistry, Kitasato University School of Medicine, 1-15-1 Minami-ku, Kitasato, Sagamihara City, Kanagawa 252-0374, Japan; ^3^Shonan University of Medical Sciences Research Institute, Nishikubo 500, Chigasaki City, Kanagawa 253-0083, Japan

## Abstract

**Background:**

Previous studies suggest the presence of an association of vascular endothelial growth factor (VEGF) with osteoarthritis (OA) severity and pain in patients with knee OA. VEGF expression in human synovial fibroblasts (SFs) is induced by transforming growth factor-beta (TGF*β*). However, the signaling pathway governing TGF*β*-mediated regulation of VEGF in SFs has not been identified.

**Methods:**

OA patients who underwent total knee arthroplasty had their synovial tissue (SYT) extracted and the constituent SFs cultured. The cells were stimulated with culture medium (control), human recombinant TGF*β* (hrTGF*β*), hrTGF*β* + ALK5 inhibitor SB505124, hrTGF*β* + transforming growth factor activating kinase 1 (TAK1) inhibitor (5Z)-7-oxozeaenol, or hrTGF*β* + p38 inhibitor SB203580 for 6 h. *VEGF* mRNA expression in SFs was examined using real-time polymerase chain reaction and VEGF protein production in the cell supernatant was examined using enzyme-linked immunosorbent assay. Additionally, phosphorylated levels of SMAD2 and p38 were examined using western blotting.

**Results:**

ALK5 (SB505124) and TAK1 (5Z-oxozeaenol) inhibitors completely suppressed TGF*β*-induced *VEGF* mRNA expression and VEGF protein production. Both SB505124 and 5Z-oxozeaenol also suppressed SMAD2 and p38 phosphorylation. The p38 inhibitor (SB203580) partially inhibited TGF*β*-mediated *VEGF* mRNA and VEGF protein production.

**Conclusion:**

TGF*β*-mediated regulation of *VEGF* expression and VEGF protein production in the SYT of OA patients occurs through both the canonical and noncanonical pathway.

## 1. Introduction

Recent evidence indicates that the vascular endothelial growth factor (VEGF) plays a role in human knee osteoarthritis (OA) pathology. Elevated levels of VEGF in OA joints have been observed in the synovial tissue (SYT) [1–4], synovial fluid (SF) [5–8], subchondral bone [9], and articular cartilage [10, 11]. In addition, increased levels of VEGF expression are reportedly linked to greater OA severity [7, 12] and pain [13, 14]. VEGF levels in SF are positively correlated with Kellgren/Lawrence (KL) grade, with higher levels observed in KL4 than KL2 patients [7]. Furthermore, OA patients with a visual analog scale (VAS) score for pain ≥6 have significantly higher synovial VEGF mRNA expression than those with VAS < 6 [14]. Based on these findings, a deeper understanding of the mechanisms governing VEGF regulation may be useful for identifying pharmacologic targets for OA. However, VEGF regulation in the SYT of OA patients has not been elucidated.

Recent reports have implicated TGF*β* signaling in osteoarthritic SYT in OA progression because TGF*β* levels are elevated in the SF of OA patients [15, 16]. A previous study reported that TGF*β* induced VEGF expression in vitro in synovial fibroblasts derived from rheumatoid arthritis patients [17]. However, the responsible signaling pathway was not fully elucidated.

TGF*β* signaling occurs via canonical and noncanonical pathways. The canonical TGF*β* signaling pathway is initiated when three TGF*β* isoforms bind the type II receptor (T*β*RII), which leads to phosphorylation of a type I receptor (T*β*RI) [18]. The phosphorylated T*β*RI, usually ALK5, subsequently transduces the TGF*β* signal through the cell to lead to phosphorylation of R-Smads. Meanwhile, noncanonical TGF*β* signaling occurs through TGF*β*-activated kinase 1 (TAK1), a mitogen-activated protein kinase (MAPK) kinase kinase (MAPKK-K), leading to activation of the p38 and Jun N-terminal kinase pathway.

Here, we investigated VEGF regulation by the canonical and noncanonical TGF*β* pathways in synovial cells obtained from the osteoarthritic synovium.

## 2. Materials and Methods

### 2.1. Patients

Human SYT was collected under the approval of the Institutional Review Board (IRB) of Kitasato University (IRB #B13-113). During total knee arthroplasty for OA, 27 suprapatellar SYT (suprapatellar pouch) samples were harvested from patients (KL grades 3 and 4). Patient background information is shown in Supplementary [Supplementary-material supplementary-material-1]. All patients provided informed consent to participate in the study one day before surgery. Once harvested, the SYT was immediately minced and dissociated by treating with collagenase solution (Sigma, St. Louis, MO) in *α*-minimal essential media (MEM) for 120 min at 37°C. The digested tissue was subsequently passed through a cell strainer with 100 *μ*m pores. After centrifugation, the cells were again suspended in culture medium comprising *α*-MEM with 10% fetal bovine serum.

### 2.2. Cell Culture

The extracted cells were seeded in six-well plates and incubated in cell culture medium for 7 days, during which time the medium was replaced twice. Flow cytometric analysis showed that more than 90% of the extracted cells were fibroblasts (CD14-CD90+) (Supplementary [Supplementary-material supplementary-material-1]). Our preliminary experiments indicated that 10 ng/ml human recombinant TGF*β* (hrTGF*β*) significantly increased *VEGF* mRNA expression compared to 1 ng/ml hrTGF*β* and culture medium (vehicle) (Supplementary [Supplementary-material supplementary-material-1]). Therefore, 10 ng/ml hrTGF*β* was used for stimulation experiments. After the 7-day incubation, synovial fibroblasts (SFs) from 8 patients were stimulated with culture medium (vehicle), hrTGF*β* (10 ng/ml), or hrTGF*β* + SB505124 (ALK5 inhibitor (ALK5i), 5 *μ*M). Meanwhile, cells from another 8 patients were stimulated with culture medium (vehicle), hrTGF*β*, or hrTGF*β* + 5Z-7-oxozeaenol (TAK1 inhibitor (TAK1i), 1 *μ*M). Finally, cells from the last 8 patients were stimulated with culture medium (vehicle), hrTGF*β*, or hrTGF*β* + SB203580 (p38 inhibitor: p38i, 1 *μ*M). All stimulations were performed for 6 h, after which total RNA was extracted from the cells and analyzed using real-time PCR, and VEGF protein levels in the cell culture supernatant were analyzed using an enzyme linked immunosorbent assay (ELISA). Inhibitors of the canonical and noncanonical pathways had no effect on cell viability at these concentrations for 6 h.

### 2.3. Real-Time PCR Analysis

After stimulation of SFs, 1 ml of TRIzol™ Reagent (Thermo Fisher Scientific, Tokyo, Japan) was added to the 6 wells and mixed thoroughly by pipetting. After homogenizing the sample with TRIzol™ Reagent, total RNA was extracted according to the manufacturer's protocol. Total RNA (1 *μ*g) was reverse transcribed using the Superscript III First-Strand Synthesis system (Thermo Fisher Scientific, Tokyo, Japan). Primers for *GAPDH* and *VEGF* were synthesized by Hokkaido System Science Co., Ltd. (Sapporo, Japan) based on primer sequences used previously [14]. Relative mRNA expression levels of *VEGF* were evaluated using real-time PCR (CFX-96®, Bio-Rad, Richmond CA, USA) with SYBR Green (TB Green™ Premix Ex Taq™ II, Takara Bio Inc, Shiga, Japan). mRNA expression was normalized to the *GAPDH* expression level using the delta-delta CT method. Relative expression was calculated using the mean of all vehicle samples.

### 2.4. ELISA

To analyze the effect of rhTGF*β* on VEGF production, the collected culture supernatant was analyzed using a commercial ELISA kit (Quantikine® ELISA Human VEGF Immunoassay, R&D Systems, Minneapolis, MN, USA) according to the manufacturer's instructions. In brief, 200 *μ*l of standard or culture supernatant was added to a primary antibody-coated 96-well plate and incubated for 2 h at room temperature (RT). The wells were then washed three times with wash buffer, and the samples were incubated with 200 *μ*l of horseradish peroxidase-conjugated secondary antibody for 2 h at RT. After three washes, 200 *μ*l of tetramethylbenzidine solution was added to the wells and the samples were incubated at RT. After 20 min, the reaction was stopped by adding sulfuric acid solution, and the absorption was measured using an ELISA reader at 450 nm with lambda correction at 570 nm.

### 2.5. Western Blotting

The mechanism by which VEGF is regulated by TGF*β* was examined by investigating phosphorylated levels of SMAD2 and p38 using western blotting. Cells from an additional 3 patients were stimulated with culture medium (vehicle), hrTGF*β*, hrTGF*β* + ALK5i, or hrTGF*β* + TAK1i for 30 min. Subsequently, cells were lysed using sodium dodecyl sulfate (SDS) sample buffer. Proteins in the cell lysates (5 *μ*g) were separated using SDS polyacrylamide gel electrophoresis and then electrophoretically transferred to a polyvinylidene difluoride membrane in blotting buffer. The membrane was blocked with 10% nonfat milk in Tris-buffered saline containing 0.05% Tween 20 (TBS-T) for 1 hour at RT. After blocking, the membrane was reacted with anti-SMAD2 rabbit monoclonal antibody (1 : 1000; cat no. #5339, Cell Signaling Technology, Inc., Danvers, MA, USA), anti-p38 MAPK (1 : 1000; cat no. #9212, Cell Signaling Technology), anti-phosphor-SMAD2 (Ser465/467) rabbit monoclonal antibody (1 : 10000; cat no. #18338, Cell Signaling Technology), or anti-phospho-p38 MAPK (Thr180/Tyr182) (1 : 1000; cat no. 9211, Cell Signaling Technology) for 1 hour at RT and subsequently with HRP-conjugated goat anti-rabbit IgG antibody for another hour. After rinsing 3 times with TBS-T, enhanced chemiluminescent detection of the proteins on the membrane was performed using the ImageQuant LAS-4000mini (Fuji Photo Film Co).

### 2.6. Statistical Analysis

Bonferroni's multiple comparisons test was used to compare differences between vehicle control- and hrTGF*β*-treated cells in Statistical Package for the Social Sciences (SPSS) (version 25.0, IBM, NY, USA). Statistically significant differences were defined by probability values (*P* values <0.05), and all statistical analyses were two-sided.

## 3. Results

### 3.1. ALK5 Inhibitor Suppresses TGF*β*-Induced Increases in *VEGF* Expression and VEGF Protein Production in Cultured SFs

**Figure 1:**
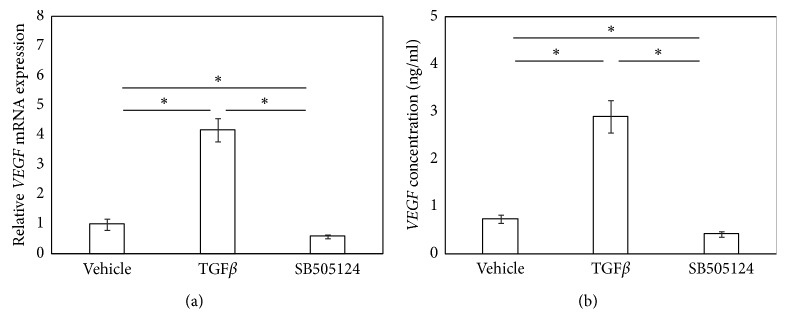
ALK5 inhibitor suppressed TGF*β*-induced *VEGF* mRNA expression and VEGF protein production. VEGF mRNA by RT-PCR (a) and VEGF protein concentration by ELISA (b). Synovial fibroblasts were stimulated with *α*-MEM (control), hrTGF*β* (TGF*β*), or hrTGF*β* + SB505124 (TGF*β* + SB505124) for 6 hours prior to RT-PCR or ELISA. Values represent mean ± SE (*n* = 8). ^*∗*^*p* < 0.05 compared to control.

Stimulation of SFs with hrTGF*β* for 6 h significantly increased *VEGF* mRNA expression (*P* < 0.002; Figure 1(a)), and exposure to the ALK5i completely abolished this increase (*P*=0.001; Figure 1(a)). Stimulation with hrTGF*β* for 6 h increased supernatant VEGF protein levels (*P*=0.001; Figure 1(b)), and exposure to the ALK5i completely abolished this increase (*P*=0.001; Figure 1(b)).

### 3.2. TAK1 Inhibitor Suppresses TGF*β*-Induced Increase in *VEGF* mRNA Expression and VEGF Protein Production in SFs

**Figure 2:**
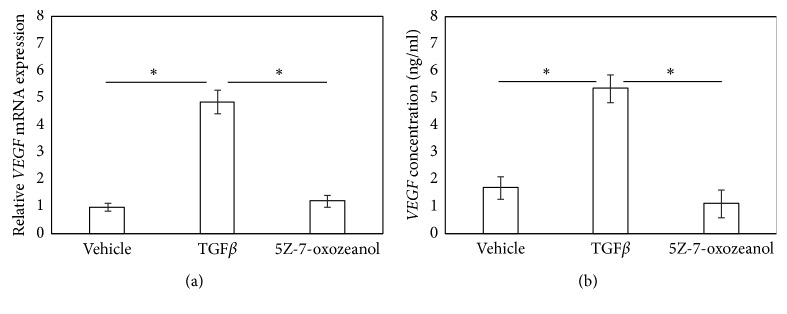
TAK1 inhibitor suppressed TGF*β*-induced *VEGF* mRNA expression and VEGF protein production. VEGF mRNA by RT-PCR (a) and VEGF protein concentration by ELISA (b). Synovial fibroblasts were stimulated with *α*-MEM (control), hrTGF*β* (TGF*β*), or hrTGF*β* + 5Z-7-oxozeaenol (TGF*β* + 5Z) for 6 hours prior to RT-PCR or ELISA. Values represent mean ± SE (*n* = 8). ^*∗*^*p* < 0.05 compared to control.

Stimulation of SFs with hrTGF*β* for 6 h significantly elevated *VEGF* mRNA expression and supernatant VEGF protein levels (*P*=0.002 and *P*=0.015, respectively; Figures 2(a) and 2(b)), and exposure to a TAK1i significantly reduced this elevation back to control levels (*P*=0.004 and *P*=0.015, respectively; Figures 2(a) and 2(b)).

### 3.3. ALK5 and TAK1 Inhibitors Suppress Phosphorylation of SMAD2 and p38

**Figure 3:**
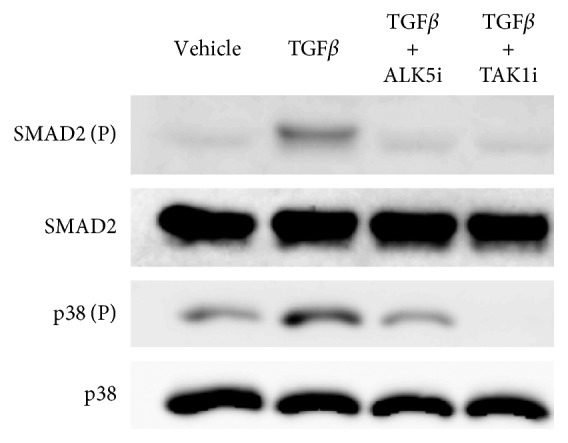
ALK5 and TAK1 inhibitors suppressed TGF*β*-induced phosphorylation of SMAD2 and p38. Western blotting was used to examine levels of phosphorylated SMAD2 and p38 MAPK. Synovial fibroblasts were stimulated with *α*-MEM (control), hrTGF*β* (TGF*β*), hrTGF*β* + SB505124 (TGF*β* + SB505124), or hrTGF*β* + SB203580 (TGF*β* + SB230580) for 30 min prior to protein extraction and western blotting. P indicates the phosphorylated form.

Given that both the ALK5i and TAK1i completely suppressed TGF*β*-induced *VEGF* expression and VEGF protein production, we subsequently examined the effect of the ALK5i and TAK1i on the phosphorylation of SMAD2 and p38. While the ALK5i completely suppressed phosphorylation of both SMAD2 and p38 (Figure 3), the TAK1i partially suppressed SMAD2 phosphorylation and completely suppressed p38 phosphorylation (Figure 3).

### 3.4. p38 Inhibitor Reduces TGF*β*-Induced *VEGF* Expression and VEGF Production in SFs

**Figure 4:**
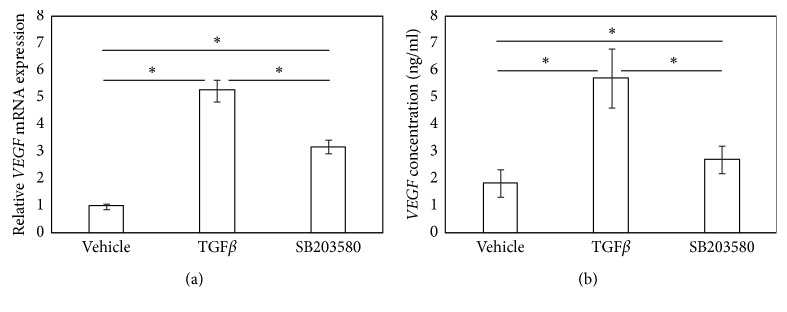
p38 inhibitor partially suppressed TGF*β*-induced *VEGF* mRNA and completely suppressed VEGF protein production. VEGF mRNA by RT-PCR (a) and VEGF protein concentration by ELISA (b). Synovial fibroblasts were stimulated with *α*-MEM (control), human recombinant (hr) TGF*β* (TGF*β*), or hrTGF*β* + SB203580 for 6 hours prior to RT-PCR or ELISA. Values represent mean ± SE (*n* = 8). ^*∗*^*p* < 0.05 compared to control.

Given that both ALK5i and TAK1i completely suppressed TGF*β*-mediated phosphorylation of p38, we subsequently investigated whether a p38 inhibitor suppresses TGF*β*-mediated VEGF production. Exposure of SFs to a p38i significantly reduced hrTGF*β*-induced *VEGF* expression and VEGF production (*P* < 0.001 and *P*=0.020, respectively; Figures 4(a) and 4(b)). However, mRNA and protein levels of VEGF remained significantly elevated compared to that observed for vehicle-treated cells (*P* < 0.002 and *P*=0.021, respectively; Figures 4(a) and 4(b)).

## 4. Discussion

This study investigated the mechanisms by which *VEGF* is regulated in the SYT of OA patients. The ALK5i and TAK1i completely suppressed TGF*β*-induced *VEGF* mRNA expression and VEGF protein production. ALK5i suppressed phosphorylation of both SMAD2 and p38, and the p38i partially inhibited TGF*β*-mediated *VEGF* expression and VEGF production. Our results suggest that TGF*β* regulates VEGF production in the SYT of OA patients via both the canonical and noncanonical pathways.

Several studies have reported that the canonical TGF*β* pathway plays a key role in regulating VEGF production [19, 20]. The ALK5 inhibitor SD-208 inhibits TGF*β*-induced *VEGF* expression with inhibition of SMAD2 phosphorylation in glioma cell lines [20]. Furthermore, the ALK5i SB505124 inhibits VEGF production in esophageal fibroblasts [19]. Similarly, we showed that SB505124 completely inhibited *VEGF* mRNA and VEGF protein production with inhibition of SMAD2 phosphorylation in SYT from OA patients. These results suggest the importance of the canonical pathway in VEGF production in osteoarthritic SYT.

Canonical TGF*β* signaling pathways include the SMAD2/3 and SMAD1/5/8 pathways, and specific inhibition of SMAD2, SMAD3, or SMAD5 by siRNA has been shown to reduce TGF*β*-mediated VEGF expression in a ganglioma cell line [20]. Furthermore, SB505124 and 5Z-7-oxozeaenol inhibit both SMAD2 and SMAD1/5 phosphorylation [19]. Therefore, whether TGF*β*-mediated VEGF production is dependent on SMAD1/5/8 or SMAD2/3 remains to be determined. In addition, SMAD2/3 and SMAD1/5/8 pathways are differentially activated depending on the active TGF*β* concentration. Further investigation of SMAD-specific silencing and the dose-dependent pathway are needed to reveal the details of canonical pathway-mediated VEGF production.

Previous studies have also proposed that TGF*β* induces VEGF expression via the noncanonical, MAPK pathway [21, 22]. A previous study showed that TGF*β* induced VEGF production in the mouse osteoblastic cell line, MC3T3E1, via p38 [21]. Inhibitors of p38 reduced TGF*β*-induced VEGF production in the rat aortic smooth muscle-derived cell line A10 [22]. However, other studies have suggested that there may be crosstalk between the canonical and noncanonical pathways [23, 24]. The ALK5i SB505124 suppresses TGF*β*-mediated phosphorylation of p38 by inhibiting ALK5 activity [23]. A TAK1i also suppressed TGF*β*-mediated SMAD2 phosphorylation in cow chondrocytes [24]. Similarly, we showed that both the TAK1i 5Z-oxozeaenol and the ALK5i SB505124 suppressed TGF*β*-mediated phosphorylation of SMAD2 and p38 in SCs. That the p38 inhibitor only partially suppressed TGF*β*-induced *VEGF* mRNA and protein production suggests that TGF*β*-induced VEGF production in the SYT of OA patients occurred through both the canonical and noncanonical pathways.

Previous studies have reported that neutralization of VEGF inhibits OA progression and pain [25, 26]. Bevacizumab, an anti-VEGF antibody, inhibited OA progression in a rabbit anterior cruciate ligament transection OA model [25]. In addition, blocking VEGF signaling using a VEGF receptor 1 antibody attenuated osteoarthritic pain in a mouse OA model [26]. Our findings on TGF*β*-mediated VEGF regulation in SYT may be useful for the future development of treatments for OA pathology.

## Data Availability

The data used to support the findings of this study are available from the corresponding author upon request.

## Supplementary Materials

Supplementary Materials Supplementary Figure 1: analysis of cell population in synovial fibroblast culture. Supplementary Figure 2: effect of TGF*β* concentration on VEGF expression. Supplementary Table 1: patient information.
