# Supplementary material for: Vascular Endothelial Growth Factor Is Regulated by the Canonical and Noncanonical Transforming Growth Factor-β Pathway in Synovial Fibroblasts Derived from Osteoarthritis Patients
Source: Biomed Res Int. 2019 Sep 29;2019:6959056. doi: 10.1155/2019/6959056 (PMC6791277; doi:10.1155/2019/6959056)
Supplement: Supplementary Materials — Supplementary Figure 1: analysis of cell population in synovial fibroblast culture. Supplementary Figure 2: effect of TGFβ concentration on VEGF expression. Supplementary Table 1: patient information. [file 6959056.f1.pdf]

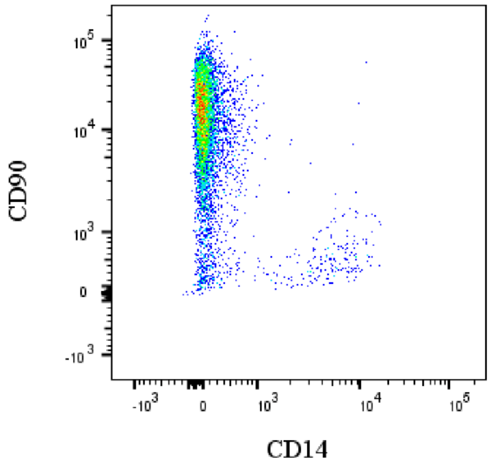

Supplementary Figure 1. Analysis of cell population in synovial fibroblast culture

Cultured synovial fibroblasts were analyzed by flow cytometry. Dot-plot analysis showed that more than 90% of the cells were CD90 + CD14- (fibroblasts).

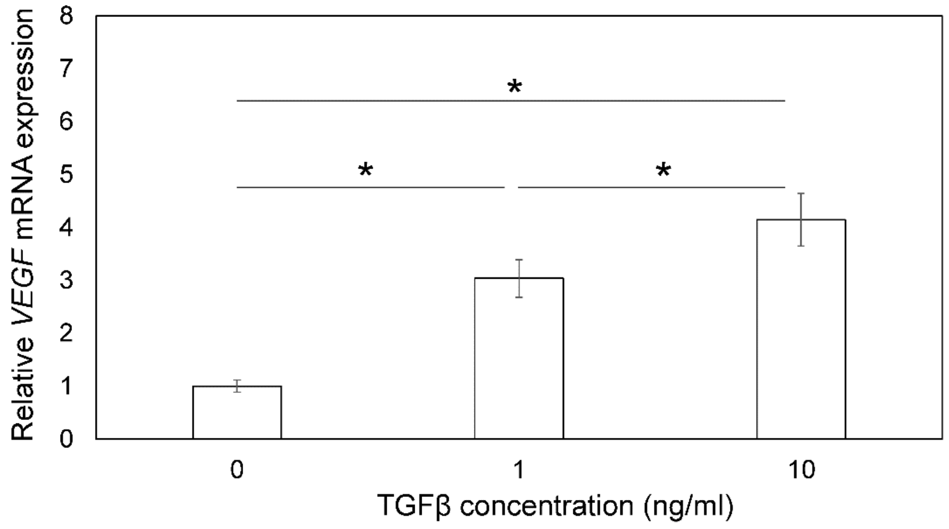

Supplementary Figure 2. Effect of TGFβ concentration on *VEGF* expression

Synovial fibroblasts were stimulated with culture medium (vehicle), 1, and 10 ng/ml human recombinant TGFβ for 6 h. After stimulation, VEGF expression was evaluated using RT-PCR. RT-PCR analysis indicated that 10 ng/ml human recombinant TGFβ (hrTGFβ) significantly increased VEGF mRNA expression compared to 1 ng/ml hrTGFβ and culture medium (vehicle) Values represent mean ± SE (n = 8). *$p < 0.05$

Supplementary Table 1. Patient information

Experiment	Effect of ALK5i on VEGF production (n=8)	Effect of TAK1i on VEGF production (n=8)	Effect of p38i on VEGF production (n=8)	Effect of ALKi and TAK1i on phosphorylation of SMAD2 and p38 (n=3)	P value
Age (y)	73.9 ± 10.3	68.1± 13.2	69.9± 8.3	61.7± 6.7	P=0.370
Male/Female, (n)	2/6	1/7	1/7	0/3	P=0.912
BMI (kg/m^2)	26.4 ± 3.9	29.0 ± 6.8	27.3 ± 5.3	28.0 ± 6.9	P=0.833

Data are mean ± standard deviation unless otherwise indicated. Continuous and categorical variables were statistically analyzed using ANOVA and Fisher's exact test, respectively. There were no differences in age, gender, or BMI among the experimental groups.

ALKi, ALK5 inhibitor; TAK1i, TAK1 inhibitor; p38i, p38 inhibitor; BMI, body mass index.
